# Personalized home based neurostimulation via AI optimization augments sustained attention

**DOI:** 10.1038/s41746-025-01744-6

**Published:** 2025-07-29

**Authors:** Roi Cohen Kadosh, Delia Ciobotaru, Malin I. Karstens, Vu Nguyen

**Affiliations:** 1 School of Psychology, University of Surrey, Guildford, UK; 2 Cognite Neurotechnology Ltd, Oxford, UK; 3 Department of Experimental Psychology, University of Oxford, Oxford, UK; 4 Amazon, Adelaide, Australia

**Keywords:** Behavioural methods, Cognitive neuroscience, Computational neuroscience, Ethics, Psychology

## Abstract

Brain-based technologies for human augmentation face challenges in personalization and real-world translation. We present an AI-driven personalized Bayesian optimization algorithm that remotely adjusts neurostimulation parameters based on baseline ability and head anatomy to enhance sustained attention at home. Validated through in silico modeling and a double-blind, sham-controlled study, our approach aligns with MRI-based models and neurobiological theories, maximizing efficacy and enabling scalable, personalized cognitive enhancement and therapy in real-world settings.

Sustained attention, the ability to maintain focus over extended periods, is essential for tasks such as driving, learning, and work-related activities^1–3^. Failures in this cognitive skill can result in serious consequences, including traffic accidents and workplace inefficiencies^3,4^. Deficits in sustained attention are also linked to a wide range of neurological and psychiatric disorders, such as schizophrenia, depression, ADHD, Alzheimer’s disease, and long COVID^5–8^.

Given its critical importance, interventions to enhance sustained attention have included cognitive training, mindfulness, physical exercise, and pharmacological treatments^9^. Neurostimulation, particularly transcranial electrical stimulation (tES), has emerged as a promising method, showing potential to enhance cognitive performance with minimal side effects. However, outcomes from neurostimulation studies have been inconsistent, often due to the “one-size-fits-all” approach that neglects individual differences in brain anatomy and baseline performance^10,11^.

Two significant barriers limit the optimization and scalability of neurostimulation: personalization and ecological validity. Personalization, which involves tailoring stimulation parameters to individuals, often requires resource-intensive methods such as exhaustive parameter testing or MRI-based adjustments^12^. These approaches are impractical for large-scale applications. Ecological validity is another challenge, as most studies occur in controlled laboratory settings that poorly reflect real-world environments like homes or workplaces^13,14^. This limits the generalizability of findings and hinders real-world implementation.

We addressed these challenges by integrating artificial intelligence (AI) with neurostimulation, creating an adaptive system based on personalized Bayesian Optimization (pBO)^15^ (Fig. 1a). This approach adjusts stimulation parameters by accounting for individual differences in baseline cognitive performance and head circumference, approximating anatomical variability^12^. By accumulating data across users, pBO refines its protocols over time, optimizing outcomes for individuals and future users with similar profiles.

**Fig. 1:**
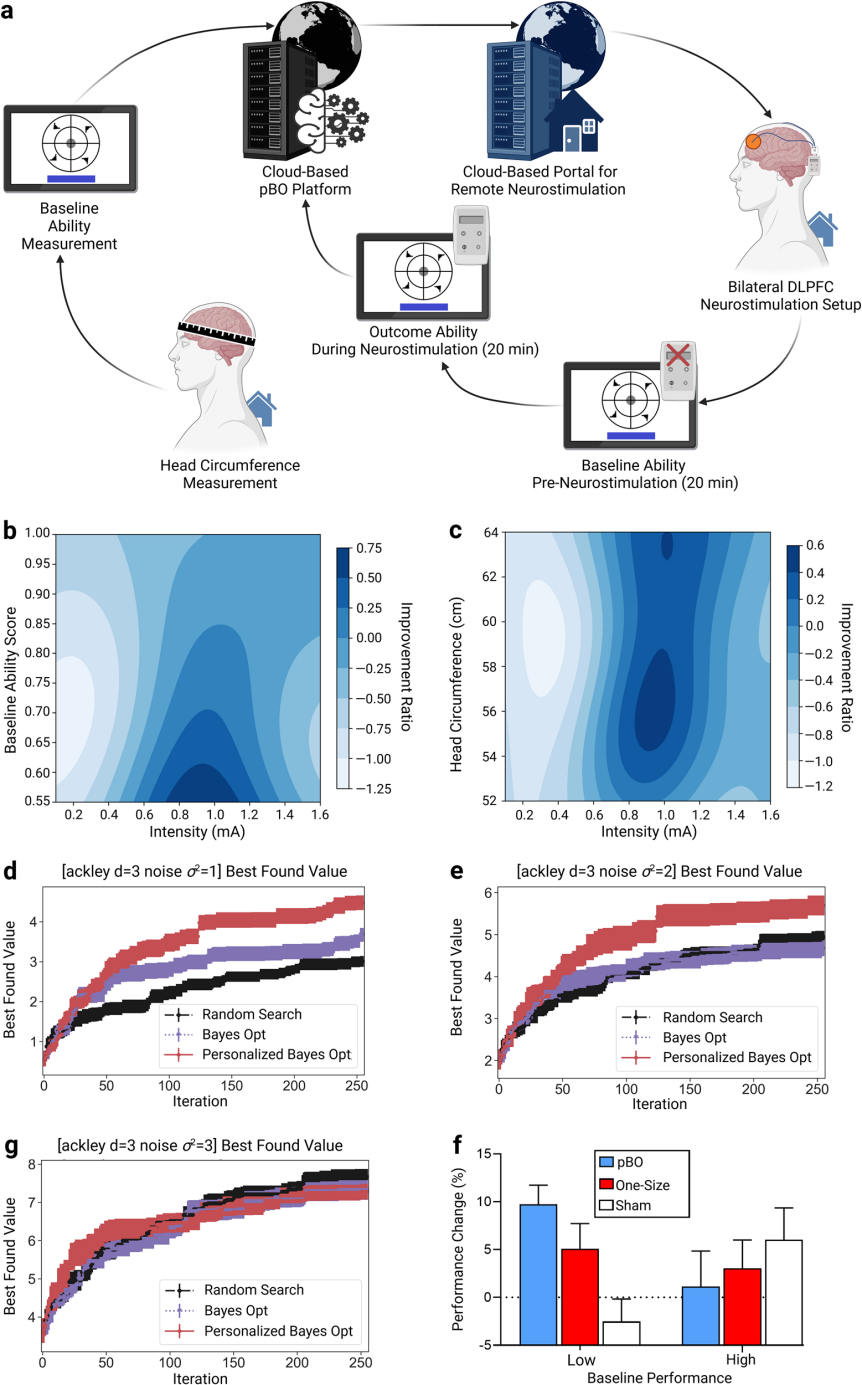
Conceptual Framework and Experimental Results. **a** Schematic of the approach: Participants received neurostimulators via courier, they measured their own head circumference, and subsequently performed a baseline sustained attention task on a tablet. These data were uploaded to a cloud-based personalized Bayesian optimization (pBO) platform, which generated individualized neurostimulation protocols to be used by a cloud-based portal for remote neurostimulation. Participants completed the 20-minute air traffic control task designed by the US Air Force Research Laboratory^37^ without stimulation, followed by 20 min of tailored neurostimulation (see Supplementary Fig. 3 and Supplementary Video). The algorithm optimized performance based on the non-parametric discrimination index (A’), defined as: $\frac{{A}^{{\prime} }{during\; neurostimulation}}{{A}^{{\prime} }{during\; Baseline}}$ and this score was fed into the pBO cloud-based platform. **b** Estimated performance (improvement ratio on the sustained attention task) plotted as a function of baseline A′ and current stimulation intensity (mA). **c** Estimated performance (improvement ratio on the sustained attention task) plotted as a function of current intensity (mA) and head circumference (cm). Performance in panels b and c is shown on a normalized scale to follow a Gaussian distribution. Lighter areas indicate lower estimated performance; darker areas correspond to regions of optimal performance. Due to high dimensionality, a direct visualization of performance as a function of A’, current intensity, and head circumference is not possible. **d–f** In silico modeling using synthetic data comparing pBO, Random Search, and non-personalized BO under noise levels σ² = 1, 2, or 3. As noise increases (panels d to f), pBO’s advantage over BO and Random Search decreases. **g** pBO-tRNS demonstrates superior performance for participants with low baseline scores. Significant neurostimulation effects were observed in low performers, while no effect was seen for high baseline performers. Y-axis represents percentage change in performance, with 0 indicating no change from baseline. Error bars represent standard error of the mean.

To improve ecological validity, our system enables home-based neurostimulation (Fig. 1a), removing logistical barriers such as travel to labs and facilitating large-scale deployment. This setting also reduces experimenter bias and increases the applicability of results to real-world conditions^14^.

In our study, healthy participants received high-frequency transcranial random noise stimulation (tRNS) in a home-based setting while completing a sustained attention task (Supplementary Fig. 1). tRNS modulates neural activity by activating sodium channels and adjusting excitation/inhibition balances^16,17^. We previously demonstrated the ability of tRNS to enhance sustained attention in a lab setting^18^. The stochastic resonance framework has postulated that specific noise level can be beneficial in non-ideal and non-linear systems, such as the brain^19^. Moreover, individuals with lower baseline performance tend to benefit the most from stimulation in the area that one wishes to augment^20^. However, it is unclear if this effect is driven by the mechanisms underlying tRNS rather than the lack of personalization.

This study consisted of three experiments:

Experiment 1: Developed a pBO algorithm to enhance sustained attention performance using tRNS.

Experiment 2: Employed in silico modeling to compare pBO against alternative optimization methods.

Experiment 3: Tested pBO-tRNS against one-size-fits-all tRNS and sham tRNS in a new sample.

In Experiment 1 we demonstrated that the pBO algorithm identified an inverted U-shaped relationship between current intensity and baseline performance (A’; a measure of the sensitivity for correctly detecting a stimulus in the task) (Fig. 1b). This supports prior findings on tRNS mechanisms, where optimal intensity lies within a “sweet spot” for maximum effectiveness^18,20,21^. The algorithm also identified intensities to avoid, which could impair performance, with both optimal and detrimental intensities depending on baseline scores.

Figure 1c reveals that higher current intensities are required with increased head circumference, following a similar inverted U-shaped pattern. Excessively low or high intensities impaired performance (brighter colors), while intermediate intensities improved it (darker colors).

In Experiment 2 we used in silico modelling to evaluate pBO against Random Search and non-personalized Bayesian Optimization (BO), utilizing the Ackley function^22^. pBO outperformed both methods, though its advantage decreased as noise levels rose, reducing parameter estimation accuracy (Fig. 1d–f; Supplementary Fig. 2a–c). The advantage of pBO diminishes in the presence of high noise, which is a general property of BO approaches^23–25^. This occurs because BO constructs a posterior belief about the unknown objective function using a Gaussian Process. When noise levels are high, it becomes more difficult for the Gaussian Process to distinguish the true underlying signal from the noise. Additionally, increased noise compels the optimizer to allocate more resources (its sampling budget) to redundant queries to reduce uncertainty.

In Experiment 1, we developed an algorithm to personalize neurostimulation parameters, identifying those enhancing performance and those requiring avoidance. Experiment 3 compared pBO-tRNS, one-size-fits-all tRNS (1.5 mA), and sham tRNS in a new sample. A mixed-effects linear regression with random effects for participant and session showed no overall effect of neurostimulation (F(2,59.57) = 0.27, *p* = 0.77). Further analysis by baseline performance (median split: 50th percentile = 0.725) revealed significant effects for low baseline performers (F(2,25.13) = 7.51, *p* = 0.003, Fig. 1g). Planned comparisons indicated pBO-tRNS outperformed sham and one-size tRNS (β = 0.76, SE = 0.29, *p* = 0.015, 95% CIs [.16,1.36]), with a better fit for a linear trend (pBO-tRNS>one-size-tRNS>sham; F(1,27.128) = 12.49, *p* = 0.001). For high baseline performers, the neurostimulation effect was not significant (F(2,25.82) = 0.56, *p* = 0.58).

Independent t-tests showed greater improvements in low vs. high baseline performers receiving pBO-tRNS (t(21) = 2.28, SE = 3.77, *p* = 0.03, 95% CIs [0.75,16.45], Cohen’s d = 0.95), but no difference for one-size tRNS (*p* = 0.61). Consistent with prior lab-based findings^18^, high baseline performers improved more than low performers under sham tRNS (t(20) = -2.01, SE = 4.2, *p* = 0.029, one-tailed, 90% CIs [−15.94, − 1.12], Cohen’s d = 0.86).

No significant differences in side effects were observed between conditions, nor were side effects predicted by tRNS intensity (Supplementary Table 1).

Our algorithm targeted A’ to optimize sustained attention without speed-accuracy trade-offs. Reaction time analysis confirmed no significant effects for either high (F(2,25.57) = 0.64, *p* = 0.54) or low (F(2,16.85) = 0.39, *p* = 0.68) baseline performers.

The results from the algorithm development (Experiment 1, Fig. 1b, c) and validation (Experiment 3, Fig. 1g), demonstrate that the benefit of pBO-tRNS was most pronounced in participants with lower baseline performance. Conversely, pBO-tRNS did not significantly enhance performance in individuals with high baseline performance (cf Supplementary Discussion). These findings align with previous research suggesting that tRNS is most effective in suboptimal conditions^18,20^. These results are in line with mechanistic explanations for tRNS, such as stochastic resonance^20^ and neuronal excitation/inhibition^17^ that predict a curvilinear neurostimulation effect on behavior. The combination of tRNS with pBO allows us to demonstrate the benefits of tRNS for those with suboptimal performance. This finding is likely to be due to the general properties of tRNS itself, rather than being the result of specific stimulation parameters. Beyond this theoretical contribution, these findings alleviate ethical concerns that neurostimulation might increase the mental gap between individuals^26^. At least with tRNS, neurostimulation may assist in closing the mental gap caused by biological or environmental constraints.

Sustained attention is closely linked to the noradrenaline system^27^. Previous research suggests that peripheral nerve activation, such as trigeminal nerve stimulation, can modulate this system. Future studies should investigate whether the effects observed in our study stem from unintended stimulation of the trigeminal nerve, as suggested for other neurostimulation protocols^28,29^. However, unlike trigeminal nerve stimulation, tRNS in this study and others^30^ did not produce adverse side effects^31^. In addition, tRNS over the dlPFC has demonstrated a larger and more enduring effect on attention-deficit/hyperactivity disorder symptoms in children compared to trigeminal nerve stimulation, suggesting at least partially distinct mechanistic pathways^32–34^.

The algorithm we developed determined that a higher current intensity is needed as a function of head size. Previous studies have suggested that head size is an important factor that can influence the amount of current that can reach the neural tissue^12^. However, current approaches for defining the required dose rely on high-resolution MRI scans for each participant, followed by further modelling to compare differences in electric field intensity. These labor- and resource-intensive procedures prevent the scalability of neurostimulation. Moreover, even after implementing these procedures, the results are still agnostic as to the optimal dose needed to maximize the benefit of neurostimulation. In contrast, the pBO algorithm aims to personalize the neurostimulation protocol to maximize the output, in this case, task performance, while considering individual characteristics that can impede optimal outcomes, such as head circumference. Therefore, this approach offers a faster, more elegant, and easier solution.

This work advances the United Nations’ Sustainable Development Goals (SDGs)—specifically SDG3 (Good Health and Well-Being) and SDG10 (Reduced Inequalities). By developing an AI-driven, home-based neurostimulation system, this research promotes accessible cognitive enhancement while addressing disparities in learning and cognitive function. Future implementations must consider ethical concerns, including safety protocols, data privacy, and equitable access to neurostimulation technology.

The study highlights the challenges of online, remote testing on monotonous tasks. Although we initially planned to enhance participant motivation with performance-based rewards, ethical concerns did not allow that. This resulted in excluding participants with non-reliable performance (see Methods, and Experiment 2 for the potential effect on pBO performance). Despite these challenges, our approach and findings demonstrate the benefits and safety of combining AI-based personalized remote neurostimulation in a real-world setting. This advancement could foster our mechanistic understanding of neurostimulation and revolutionize its use by increasing accessibility and effectiveness. In turn, this could open new avenues for enhancing cognitive performance in everyday environments for healthy individuals and for treating brain-based disorders.

## Methods

### Experiment 1’s participants

A total of 103 participants aged 18 to 35 years (*M* = 26.8, *SD* = 3.8) successfully completed at least one out of three neurostimulation sessions. Two participants could not complete any session due to failed Wi-Fi connectivity. The primary reasons for incomplete sessions were technical difficulties in executing the experimental task or unexpected scheduling conflicts. Notably, there were no dropouts related to the participants’ direct experience with the neurostimulation. As a result of these issues, 23 data points could not be successfully collected, which yielded a total of 290 neurostimulation sessions that were used for the algorithm development.

Participants were living in the UK at the time of the experiment. We excluded participants if they were pregnant, or currently on medication for any acute or chronic disorders including epilepsy or a history of epilepsy in a first-degree relative. Individuals with severe scalp skin lesions, such as unhealed wounds, recent scar tissue, or broken skin, were excluded. Those who had undergone brain surgery and had a hearing device, or any implanted metal or electronic objects in the upper part of their body, like a pacemaker, were also ineligible to participate.

Participants were recruited through advertisements placed on social media platforms such as Twitter, Facebook, LinkedIn, and Instagram. Additionally, flyers in public spaces and presentations were employed to attract potential participants. To initiate the recruitment process, individuals interested in the study contacted the study investigator using the details provided in the advertisements. The research staff then provided the Participant Information Sheet, the informed consent form, and details about the Ministry of Defence (MoD) no-fault compensation scheme. This was followed by an opportunity for the participants to clarify any queries via email or during a phone call with a member of the research team. Prospective participants expressing their willingness to join the study were instructed to return the signed consent form at least 24 h after receiving the Participant Information Sheet and associated documents. The study was approved by the UK MoD Research Ethics Committee (MODREC). Supplementary Fig. 1 presents a picture of one of the participants. The authors have obtained written consent to publish the image.

### Experimental procedure

At the beginning of the first session, participants measured their head circumference at home using the measuring tape provided by the research staff and input the results when prompted on the tablet. The measurement was repeated three times and the average of the three was computed to minimize measurement errors. The participants also completed the Eligibility Questionnaire. In addition, the participants also completed the Cognitive Failures Questionnaire, the Mind-Wandering Questionnaire^35^ and the Brief State Rumination Inventory^36^. The data from these questionnaires is not the focus of the current report, and it is therefore not reported here.

### Sustained attention task

During all sessions, participants performed the air traffic controller task used by the US Air Force Research Laboratory^37^ which was programmed in PsychoPy®.

This task was chosen as a lab-based tool to capture vigilance and cognitive control performance via the detection of rare events, similar to professional operator tasks^37–41^.

In a previous lab-based experiment (Malin I. Karstens, unpublished PhD dissertation) using the same task, we found an acceptable test-retest reliability (α = 0.7, *p* < 0.001, *n* = 39) based on 10-minute baseline blocks measured on consecutive days. This aligns with prior research demonstrating the reliability of similar go/no-go tasks^42^ and indicates its ecological validity via links to other everyday life measures of attention^38,43^. In this task, the user needs to detect an infrequent event (11.1% of all trials) in which one aircraft out of four deviates from the “safe path” condition, indicating an imminent collision (Supplementary Fig. 3a, and Supplementary Video). In such cases where a “critical” event is detected, the participant must refrain from pressing a blue button on the display (Supplementary Fig. 3b). Otherwise, the participant pressed the blue button in each “safe path” event. Except for the blue button, all images were in greyscale to control for color blindness. Participants were advised to keep their hand close to the table without touching it, maintaining a posture that was comfortable yet ready for minimal movement, as each trial lasted a maximum of two seconds. Only new touches > 150 ms after the stimulus display were counted in each trial, as touches before 150 ms are likely premature responses or late responses to the previous trial since the new stimulus cannot be processed that quickly.

The initial session included time for viewing instructions and practicing the task without brain stimulation. In Sessions 2, 3, and 4, participants completed the task with the self-administered neurostimulation using the provided headgear (Supplementary Fig. 3c). Instructions for setting up the headgear were provided, and a researcher was available to answer any questions both beforehand and during the sessions.

In Session 1, participants completed a practice period of 12 min consisting of 1 min of slow practice (each stimulus was shown for 3 s, with a 1-second inter-stimulus interval consisting of the radar being shown without the airplanes); 1 min of medium speed (each stimulus was shown for 2 s, with a 1-second inter-stimulus interval consisting of the radar being shown without the airplanes), and 10 min of normal speed (each stimulus was shown for 1 s, with a 1-second inter-stimulus interval consisting of the radar being shown without the airplanes) followed by a baseline session of 10 min. During the practice session, participants received visual feedback for correct and incorrect decisions. In total, Session 1 lasted 30 min and the neurostimulation headgear was not worn during this session. Following the completion of Session 1, participants scheduled the next sessions on a call with the research staff.

Sessions 2-4 lasted approximately 1 h 30 min and were completed within a maximum of one week. The participants completed the air traffic controller task for 20 min (two blocks of 10 min of normal speed trials) without neurostimulation and then for 20 min (two blocks of 10 min of normal speed trials) whilst receiving neurostimulation. We included a break of 1 min between all blocks.

Although the 10-minute task blocks were shorter than real-world attention-demanding activities, this duration optimized participant engagement while maintaining experimental control. Prior research supports the ecological validity of the Sustained Attention to Response Task (SART), even with a lower number of trials^38,43^.

### Neurostimulation

Before each neurostimulation session, participants completed the Neurostimulation Eligibility Questionnaire and four additional questions concerning recent alcohol, caffeine or drug consumption, or lack of sleep. None of the participants reported having drunk more than 3 units of alcohol in the 24 h before the experiment; having drunk alcohol during the day the experiment took place; having had more than one cup of coffee or other sources of caffeine in the hour before the experiment; or having slept less than 6 h the previous night to the experiment.

Since the neurostimulation was self-administered and monitored remotely, the study participants were supplied with an internet-enabled tablet and a CE-marked tRNS headgear (HomeStim, Neuroelectrics, Barcelona). The internet allowed updating the stimulation protocol onto the tablet prior to the sessions and downloading the study data after the session was finalized but was not used while the neurostimulation was in progress. We delivered the study equipment to participants’ homes and they returned the equipment using fully tracked delivery services (DHL Group, Germany). During the experiment, the participant was required to adhere to certain conditions including:

- Sitting position on a chair.
- Placing the tablet in front of them.
- Minimizing distracting factors such as phones or other people in the same room.
- Completing the experiment at approximately the same time on 4 different days within a week.
- Stable internet connection.

Participants received step-by-step instructions on the tablet and during a video call with the research staff on how to position the cap and electrodes, and how to stop the stimulation should they decide to do so at any point. After positioning the cap and electrodes, an impedance check was performed by the device, after which the participant could initiate the neurostimulation via a start button. The battery-driven stimulator delivered the neurostimulation depending on the participant ID code. The neurostimulation parameters could not be changed by the participants, as they were pre-set by the experimenter which limits the intensity and duration of stimulation as well as the frequency of sessions. The stimulation was deactivated until the experimenters set up a single session with a pre-set duration and intensity.

We applied a bifrontal electrode montage using the international 10-20 electroencephalography system. One electrode was placed over the right dorsolateral prefrontal cortex (DLPFC, F4) and another electrode was placed over the left DLPFC (F3). The electrodes were inserted in saline-soaked surface sponges (25 cm^2^) and fixed into predefined positions on a neoprene cap that were marked by the experimenter before the equipment was sent. At the end of the session, participants rinsed the electrodes with water and put them out to dry till the next session. The data was saved automatically on the tablet and transferred to the researchers over a secure cloud-based platform.

Participants’ performance and progress were remotely monitored on the research platform to ensure compliance and prompt detection of any issues during testing. Stimulation could not commence unless the correct channels were used. Unlike transcranial direct current stimulation, which requires placement of the anode and cathode for targeted current flow, tRNS is polarity-independent^44^. As a result, swapping the assignment of the two channels between the electrodes would not affect the stimulation protocol or its efficacy. Furthermore, the platform flagged any setup issues, such as failed impedance checks, and sent automated email alerts to the research team at every stage of the setup. Stimulation would not proceed unless impedance checks were successfully passed and was automatically terminated if impedance dropped below acceptable levels or if the participant removed the cap. In such cases, research staff promptly contacted participants with additional instructions to resolve the issue.

tRNS started with a ramp-in phase and ended with a ramp-out phase, lasting 30 s each. The current was administered with an intensity from 0.1 mA up to 1.6 mA between ramp-in/-out, depending on what parameters were chosen by the algorithm. Electrode montage and stimulation parameters, including impedance, were monitored throughout the experiment and were documented automatically. After each stimulation session, the Neurostimulation Side Effects Questionnaire was completed by the participant to record any adverse events. Moreover, before starting a new session, the researchers asked the participants whether they had experienced any side effects since the last session.

The primary outcome that the algorithm aimed to optimize was the participants’ non-parametric discrimination index (A’)^37^, based on the participants’ baseline performance on this task and their head circumference. We estimated A’ by the following Eq. (1):

1 $${A}^{{\prime} }=({\rm{cumulative\; distribution\; function}}(Z({hit\; rate})-Z({false\; alarm\; rate})/{Sqrt}(2))$$

Where hit rate indicates the probability of responding yes on “safe” trials and false alarm rate indicates the probability of responding yes on “critical” trials.

The primary outcome measure reflected the change in performance and was defined as presented in Eq. (2):

2 $$\frac{{A}^{{\prime} }{during\; neurostimulation}}{{A}^{{\prime} }{during\; Baseline}}$$

Therefore, any ratio above 1 indicates augmented performance. For example, a primary outcome that equals 1.05 reflects a 5% improvement. Any ratio below 1 indicates deteriorated performance.

Overall, this phase consists of an initial sampling (burn-in) phase of 72 sessions, and a subsequent pBO stage of 218 sessions. The burn-in phase included random stimulation parameters. This burn-in stage enables the algorithm to be trained on participants’ performance on the air traffic controller task as measured by primary outcome. Subsequently, we initiated the pBO stage, where the pBO algorithm was retrained before each stimulation session to determine the optimal parameters for the upcoming session, aiming to enhance task performance as measured by the primary outcome.

Please note that the primary aim of this study was to maximize overall sustained attention performance rather than analyze moment-to-moment fluctuations. Therefore, the optimization algorithm was developed using compound scores that distinguish signal from noise (A’) in this detection task.

### pBO algorithm development

Let x denote the parameter to be optimized and p be the personalized variable. In particular, in Experiment 1: we defined the two-dimensional personalized variables p as the Head circumference and the A’ baseline score. We then defined the main variable x (to be optimized) as the Current Intensity. Similarly in Experiment 2: the personalized variable p is defined as two parameters and one main variable x.

We define the covariance function (Eq. (3))

3 $$k\left(\left[x,p\right],\left[{x}^{{\prime} },{p}^{{\prime} }\right]\right)=k\left(x,{x}^{{\prime} }\right)\times k\left(p,{p}^{{\prime} }\right)$$

where according to Eq. (4)

4 $$k\left(x,{x}^{{\prime} }\right)=\exp \left(-\frac{{\left[x-{x}^{{\prime} }\right]}^{2}}{{\sigma }_{x}}\right)$$

and Eq. (5)

5 $$k\left(p,{p}^{{\prime} }\right)=\exp \left(-\frac{{\left[p-{p}^{{\prime} }\right]}^{2}}{{\sigma }_{p}}\right).$$

The length scale parameters include ${\sigma }_{x}$ and ${\sigma }_{p}$. For example, if the personalized length-scale ${\sigma }_{p}$ is extremely large, it means the performance is not changing with respect to the personalized variable p. On the other hand, if ${\sigma }_{p}$ is small, it means the performance function is changing rapidly with the personalized variable. We later maximized the marginal likelihood to estimate these length scale parameters directly from the data following Williams and Rasmussen^45^.

Using the defined $k\left(\left[x,p\right],\left[{x}^{{\prime} },{p}^{{\prime} }\right]\right)$ in Eq. (3), we then estimated the predictive mean and predictive variance of the Gaussian process model at any value of [x,p] as follows (Eq. (6)):

6 $$\mu \left(x,p\right)=k\left(\left[x,p\right],Z\right)K{\left(Z,Z\right)}^{-1}y$$

$z=\left[x,p\right]$ and $Z={\left\{{z}_{i}\right\}}_{i=1}^{N}$ for brevity.

Using the above predictive equations, we define the acquisition function that balances these predictive mean $\mu \left(x,p\right)$ and variance $\sigma \left(x,p\right)$ to select the next point (Eq. (7)).

7 $$\alpha \left(x,p\right)=\mu \left(x,p\right)+\kappa \sigma \left(x,p\right)$$

where $\kappa$ is a constant indicating how much we want to exploit versus explore. Then, the next point is selected as in Eq. (8)

8 $${x}_{t+1}=\arg \,\max \,\alpha \left(x,p\right)$$

Note that this auxiliary maximization problem can be easily optimized by standard numerical techniques.

We ran the algorithm in the cloud using Google Cloud Compute Engine with a basic configuration of 2 CPUs and 8 GB of RAM.

### Experiment 1 Statistical analysis and sample size justification

A stopping criterion of 300 data points was set in Experiment 1, based on prior Bayesian optimization studies. Given the potential for increased data variability in human-based research, we set *n* = 100 per dimension (baseline ability, head circumference, and current intensity), exceeding prior benchmarks^15,23^. Previous research has found that A’ is a reliable non-parametric indicator of performance on the air traffic controller task, recommended for use when the data may not be normally distributed^37^. Based on our previous lab results^18^, we manipulated current intensity as a continuous variable (0.1 steps (in mA): minimum 0.1 to maximum 1.6). As the personalized variables, we included individual differences in baseline ability (A’ as assessed by the task before neurostimulation) and head circumference (which allows the approximation of the electric field^12^). Individuals’ baseline performance for the task was calculated cumulatively, as a preliminary data analysis during the burn-in phase (first 72 neurostimulation sessions) suggested that this approach allows a more accurate estimation of the baseline ability rather than basing it only on session 1. Specifically, the baseline for the first stimulation session was determined based on the performance in the baseline task conducted on a separate day. For the second stimulation session, the baseline was established as the average of two baseline performances: the initial baseline session and the first stimulation session. Finally, the baseline for the third stimulation session was derived as the average of the performances across three baseline points: the baseline session, the first stimulation session, and the second stimulation session. This approach ensured a progressively refined baseline.

### Parameter to be optimized

Our algorithm took two personalized scores as the input; baseline sustained attention and head circumference. Then, the pBO algorithm suggested the current intensity for the specific session. After a participant completed a session, the A’ from that session was automatically calculated and used as the output score to calculate the outcome—the ratio of improvement (A’ stimulation/A’ baseline)—indicating how well the participant performed. We describe the range of these parameters below based on our data:

- [Input] A’ Baseline [0.3 - 1]
- [Input] Head circumference [52 - 66] cm
- [Input suggested by pBO] Current intensity [0.1,1.6] mA
- [Output] A’ stimulation [0-1]
- [To be maximized] Ratio = A’ stimulation / A’ baseline (Outcome)

### In silico modelling using synthetic data (Experiment 2)

To compare the performance of our pBO algorithm against the Random Search and non-personalized BO (by merging all users into a *single* user and then performed the same algorithm) methods, we utilized the Ackley function^22^, which is widely used for testing optimization algorithms. The Ackley function is defined using the following Eq. (9):

9 $$f({\boldsymbol{X}})=-a\exp \left(-b\sqrt{\frac{1}{d}\mathop{\sum }\limits_{i=1}^{d}{x}_{i}^{2}}\right)-\exp \left(\frac{1}{d}\mathop{\sum }\limits_{i=1}^{d}\cos ({cx}_{i})\right)+a+\exp (1)$$

where the number of input dimensions d = 3 including one dimension for current intensity and two personalized dimensions for head circumference and baseline score.

We varied the levels of output noise (${\sigma }^{2\,}=\mathrm{1,2,3})$ in which the Gaussian noise is added to the function output as in Eq. (10)

10 $$f(x)=f(x)+\epsilon \,{\rm{w}}{\rm{h}}{\rm{e}}{\rm{r}}{\rm{e}}\,\epsilon \,\sim N(0,{\sigma }^{2})$$

### Experiment 3

We followed the same methods as in Experiment 1, with the following exceptions:

### Participants

In Experiment 3, a total of 35 participants (18 to 35 years old, *M* = 29, *SD* = 4.75) completed the three assigned sessions and two more completed their pBO and sham sessions but not the one-size session (1.5 mA), leading to a total of 37 participants (M = 28.97, SEM = 4.63).

Participants completed three double-blind conditions on different days in a counterbalanced order, receiving 1) pBO-tRNS according to the algorithm we developed in Experiment 1; 2) sham tRNS; 3) one-size 1.5 mA tRNS. See Supplementary Fig. 4 for the percentage distribution of current intensity levels sampled during the pBO-tRNS sessions. We chose 1.5 mA tRNS as the one-size-fits-all current intensity, as we recognized that studies in the field predominantly use parameters between 1-2 mA when testing human adults^20^.

To ensure an unbiased comparison, the one-size-fits-all tRNS intensity was selected independently of Experiment 1’s findings. Otherwise, selecting intensity based on the algorithm’s output could raise concerns that a literature-based intensity might yield better results than the personalized approach. For a detailed justification of the selection of 1.5 mA tRNS, please refer to the Supplementary Methods and Supplementary Figs. 5 and 6.

For sham stimulation, the current was ramped up to 1.5 mA for 30 s and then faded out over another 30 s at the beginning of the first tRNS block. This mimicked real stimulation sensations while preventing active neurostimulation. The headgear montage was identical in all conditions. Participants were unaware of the sham condition, including any differences between the conditions, to minimize expectancy effects^46^. The tRNS current intensity was implemented and uploaded by a second experimenter, who was never in contact with the participant to maintain blinding of the primary experimenter leading the sessions.

### Statistical analysis and sample size justification

For Experiment 3, sample size was determined using G*Power (version 3.1.9.6). To achieve 80% power (1-β = 0.8) at α = 0.05, a paired t-test required 28 participants for a directional hypothesis (tRNS>sham) or 35 participants for a non-directional hypothesis, based on effect size estimates from prior tRNS studies on sustained attention^18^.

To examine stimulation effects, we used linear mixed effects models, which account for within-subject correlations more optimally compared to ANOVA and handle missing values automatically, allowing maximum use of available data. We used the IBM® SPSS® software platform (version 29.0.1) to perform the linear mixed effects analysis with Restricted Maximum Likelihood (REML) on the outcome measure, with participants and sessions as the random factors, and stimulation (pBO-tRNS, one-size tRNS, and sham tRNS) as the predictor. In case of a significant result (α = 0.05), we examined the source of this effect by running comparisons between the stimulation conditions. While we do not control for the family-wise error rate, the main result in the manuscript, showing the advantage of pBO-tRNS vs. sham and one-size tRNS, survived the Bonferroni-Holm correction procedure. We confirmed that the residuals are normally distributed using the Shapiro-Wilk test. In one of the analyses, the results from this test were trending (*p* = 0.058), and an extreme outlier was identified and excluded (residual = 0.29, while Cook’s distance considers any value above.125 as highly influential). However, we also repeated the reported analyses without excluding this outlier, and the results still hold.

We used a median-split on baseline performance rather than treating it as a continuous covariate, as baseline A’ was included in the primary outcome calculation:

$$\frac{{A}^{{\prime} }{during\; neurostimulation}}{{A}^{{\prime} }{during\; Baseline}}$$

It has been shown that the inclusion of baseline performance as a covariate, as in this case, would create a statistical artefact and therefore should be avoided^47^.

Given the extremely low side effect values (see Supplementary Table 1), the participants’ lack of awareness of the different tRNS conditions, and the absence of group differences in reported side effects, there is no theoretical or empirical basis to expect any impact on the results.

### Criteria for data exclusion/inclusion

One of the main challenges of a remote cognitive experiment, which does not bring an immediate benefit to participants, is that it may lead to a lack of motivation/interest and therefore the data may be unreliable. This aspect is less of a concern when large data is collected as in Experiment 1 (103 participants, total 290 sessions). However, it is a potential concern when baseline performance was used to define the optimal parameters for the personalized intervention (see Fig. 1d–f and Supplementary Fig. 2). We therefore first examined baseline performance every day (Session 1, as well as the first 20 min of Session 2-4, which were not subjected to neurostimulation) displayed unexpected deviations in performance. We observed that in some cases the data was highly volatile, showing >20% differences in performance between the baseline sessions. Therefore, the data from these sessions (27.1%) could not allow a reliable estimate of the baseline data for the pBO (see Experiment 2) and the intervention’s outcome was excluded. A more liberal threshold than 20% baseline fluctuation would have compromised the reliability of our algorithm, as tRNS intensity was determined based on baseline performance and head circumference. Greater deviations from a participant’s true baseline would have led to inaccurate parameter estimation, reducing the efficacy of tRNS and potentially producing detrimental effects. Conversely, a stricter threshold would have resulted in an unacceptably high exclusion rate and a reduced sample size, limiting the study’s statistical power. Importantly, baseline fluctuations in performance were not driven by learning effects, as there was no significant effect of session on baseline A’ (F(2,70.74) = 0.81, *p* = 0.45). Instead, we found that higher variability in baseline performance was associated with poorer overall baseline task performance. Specifically, participants who exceeded the 20% fluctuation threshold performed significantly worse at baseline compared to those who remained within the threshold (F(1,95.72) = 17.74, *p* = 0.00006). Finally, our exclusion rate (~ 27%) is in line with similar exclusion criteria used in other fields to ensure reliable data collection^48^. However, the exclusion of these participants may limit the generalizability of our findings to individuals who demonstrate higher motivation in monotonous tasks.

Furthermore, two additional sham sessions were excluded due to a task crash during the experiment. When the excluded sessions included the sham condition, the complete data from these participants (9.3%) was excluded as it is not possible to assess the advantage of neurostimulation without the sham condition.

## Sustainable development goals (SDGs)

Our work contributes to the United Nations’ SDGs, specifically SDG3 (Good Health and Well-Being) and SDG10 (Reduced Inequalities). By advancing a personalized, home-based neurostimulation system, this research promotes mental health and cognitive well-being (SDG3) while addressing critical barriers to accessibility and scalability. The inclusion of an AI-driven, adaptive framework ensures that effective interventions can be tailored to individual needs, including those with lower baseline abilities, thereby fostering equity in cognitive enhancement. This focus on supporting individuals with suboptimal performance aligns with the goal of reducing inequalities in health outcomes and cognitive capabilities (SDG10), offering a pathway for widespread, scalable implementation that benefits diverse populations.

## Supplementary information

Supplementary information

Supplementary Movies 1

## Data Availability

While we are able to share the data from Experiments 2 and 3 upon request, please note that we cannot disclose the data from Experiment 1 (algorithm development) due to restrictions imposed by the UK Ministry of Defence, which funded this aspect of the work.
