# Supplementary material for: Personalized home based neurostimulation via AI optimization augments sustained attention
Source: NPJ Digit Med. 2025 Jul 29;8:463. doi: 10.1038/s41746-025-01744-6 (PMC12307579; doi:10.1038/s41746-025-01744-6)
Supplement: Supplementary file 1 — Supplementary information [file 41746_2025_1744_MOESM1_ESM.pdf]

Supplementary Information

Personalized Home Based Neurostimulation via AI Optimization Augments Sustained Attention

Roi Cohen Kadosh[1,2,*], Delia Ciobotaru[1,2,$], Malin I. Karstens[2,3,$], and Vu Nguyen[4**]

[1]School of Psychology, University of Surrey, Guildford, UK

[2]Cognite Neurotechnology Ltd, Oxford, UK

[3]Department of Experimental Psychology, University of Oxford, Oxford, UK

[4]Amazon, Adelaide, Australia

*Corresponding Author (r.cohenkadosh@surrey.ac.uk)

**Work done outside Amazon

[$]These authors contributed equally

Supplementary Discussion

Our findings suggest that individuals with high sustained attention capacity (e.g., air-traffic controllers) may derive limited benefit from tRNS. However, prior research indicates that tRNS effects emerge under suboptimal conditions—such as high cognitive load or fatigue[1,2]. Future studies should examine whether high-performing individuals experience benefits under cognitively demanding circumstances.

Supplementary Methods

Rational for selecting 1.5mA tRNS intensity

We selected 1.5mA based on a substantial body of tRNS literature, where 1.5mA tRNS has been widely used and shown to enhance cognitive performance and neural excitability compared to sham stimulation. These effects have been demonstrated across various domains, including perception[3-5], learning and memory[6-8], and cortical excitability[9]. While findings at 2mA tRNS are more heterogeneous, studies have also reported benefits at this level, such as enhancing attentional performance[10,11] and modulating the dorsal and ventral attention network[11], improving working memory[12], enhancing peripheral reading ability[13], and increasing cortical excitability (in this case, yielding significant effects compared to sham that were absent at 1mA and 1.5mA)[14]. Given this evidence, selecting 1mA as a fixed intensity would have disregarded key findings supporting higher stimulation levels and could have introduced its own limitations. To further validate our choice, we consulted two generative AI models (ChatGPT and Gemini) to recommend an intensity for high-frequency tRNS in the context of cognitive enhancement. Both models independently suggested an intensity of 1.5 mA (see Supplementary Figures 5–6). This convergence between existing literature and AI-generated, ostensibly unbiased recommendations provided additional confidence in selecting 1.5 mA as a reasonable

benchmark for conventional tRNS application. Importantly, this approach also ensured an unbiased comparison: the fixed-intensity tRNS condition was determined independently of the findings from Experiment 1. Selecting an intensity based on the algorithm's output could otherwise raise concerns that a literature-derived value might have produced more favourable results than the personalised approach.

Nevertheless, when we repeated this query five days later, ChatGPT recommended 1 mA, while Gemini suggested a range of 1–2 mA. In our view, this variability further underscores the fact that there is currently no clear consensus regarding whether 1 mA or 1.5 mA is optimal, thereby reinforcing the rationale for a personalised approach.

We would like to emphasise that, in our view, the observed effects of pBO-selected intensities were not solely attributable to lower stimulation levels. If reduced current intensity alone accounted for the observed improvements, one would expect consistent convergence across participants to a single optimal current intensity (e.g., 1 mA). However, as illustrated in Supplementary Figure 4, the optimised intensities exhibited substantial inter-individual variability, thereby supporting the rationale for individualised parameter selection rather than the application of a fixed, lower intensity.

While it remains an open question whether 1mA tRNS would have been more effective than 1.5 mA in a conventional setting, our findings indicate that pBO offers a more effective approach by tailoring stimulation parameters to maximise individual benefits. A one-size-fits-all paradigm is likely to be less effective for a greater proportion of individuals and may fail to provide the optimised protocol required for maximal enhancement effects.

An anonymous reviewer suggested an alternative control condition—such as permuting stimulation intensities based on the pBO distribution—to further isolate the effects of personalisation. We acknowledge the merit of this proposal and agree that such a design could be valuable in future studies following our validation of the pBO–tRNS effect. However, we also note that this approach poses methodological challenges, including the risk of inadvertently assigning stimulation intensities that are not only suboptimal but potentially detrimental at the individual level (see Figure 1b–c). Future research should address these issues to further refine personalised stimulation paradigms.

Supplementary Figure 1. Home-based neurostimulation. A participant wears the neurostimulation headgear and completes the experimental task at home. The individual in the figure provided a written permission to use this picture.

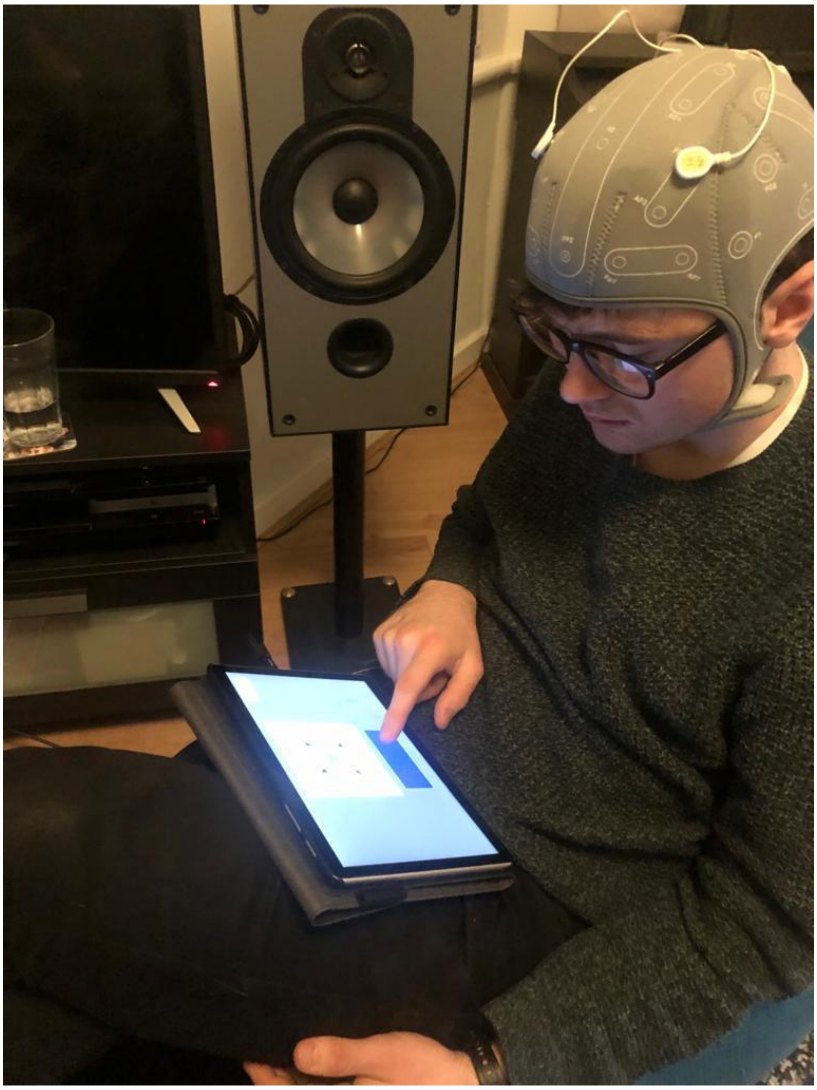

Supplementary Figure 2. *In silico* modelling using synthetic data comparing the performance of the pBO algorithm, the Random Search, and the non-personalized BO methods. We set the noise levels as $\sigma^2 = 1,2,3$ (panels a, b, and c, respectively). We illustrate the performance in terms of the Euclidean distance to the true optima location (input). We show that as the noise level increases, the efficacy of pBO versus BO and Random Search decreases. This makes sense because the large amount of noise can make the signal less informative and thus the optimization process can get confused. Therefore, the overall optimization will be degraded.

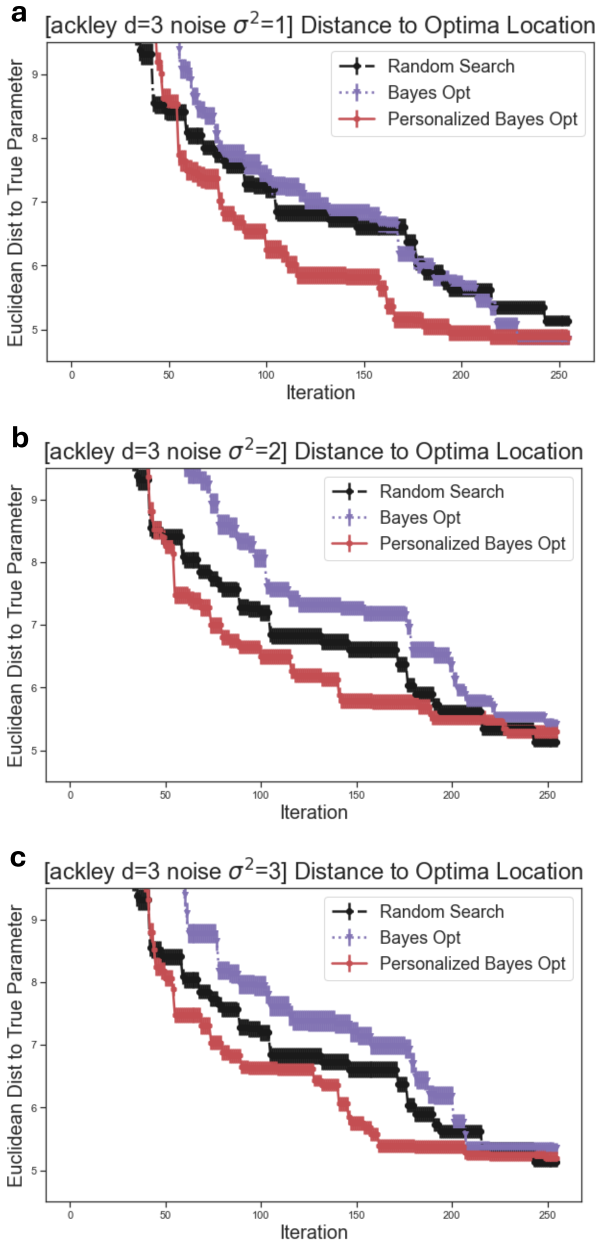

Supplementary Figure 3. The simulated air traffic controller task. a) A static aircraft symbol appeared within each quadrant of a radar circle. In the "safe path" condition, the airplanes are all oriented in the same direction, which can be either clockwise or counterclockwise. In the "critical path" condition, one of the aircrafts is oriented in the opposite direction to the other 3. **b**) A block of the task lasted 600 seconds. A stimulus is presented randomly for 1 s with a one-second inter-stimulus interval during which the radar is displayed without any airplanes. The participants were instructed to press a blue button on the tablet display only when detecting that all the aircrafts are oriented in the same direction ("safe path", left display), and avoid pressing such button when an aircraft is oriented in the opposite direction to the other three, indicating a collision is imminent ("critical path"). **c**) Day one served primarily to collect the head circumference and for the participants to familiarize themselves with the task through practice trials and the initial two baseline task blocks. The following sessions were performed with the headgear mounted and included two baseline and two tRNS task blocks.

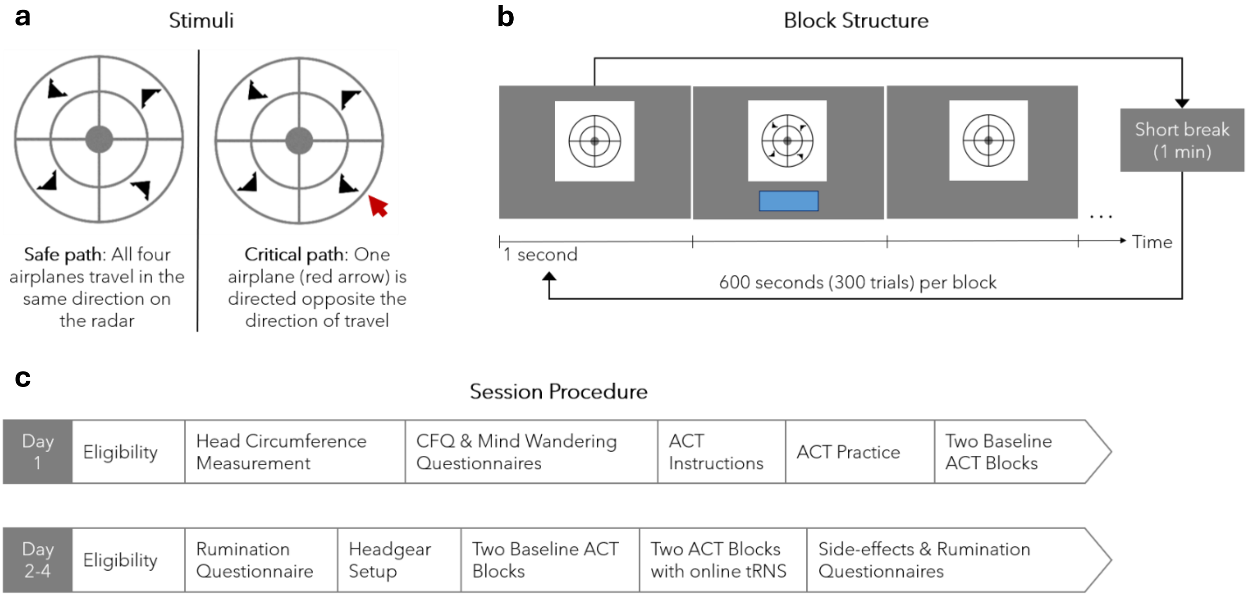

Supplementary Figure 4. Percentage Distribution of Current Intensity Sampling. This figure displays the percentage distribution of current intensity levels sampled during the pBO sessions of Experiment 3. The bar heights represent the number of occurrences for each current intensity. Descriptive statistics for current intensity are as follows: Mean = 0.781 mA, Median = 0.9 mA, SD = 0.308 mA, Min = 0.1 mA, Max = 1.5 mA, N = 37.

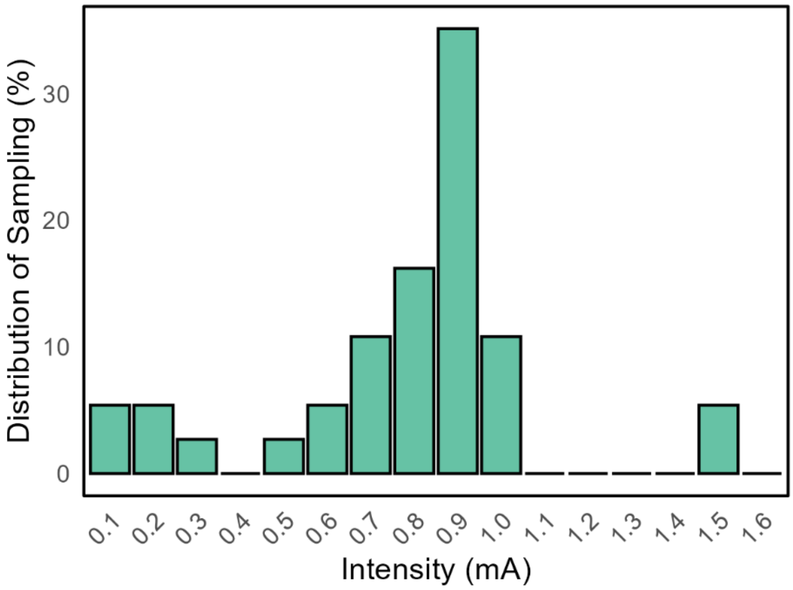

Supplementary Figure 5. The response to the prompt asking ChatGPT "I would like to use high frequency transcranial random noise stimulation for cognitive enhancement. What mA intensity do you suggest I will choose for stimulation?" yields a recommendation to use 1.5mA.

The optimal intensity for **high-frequency transcranial random noise stimulation (hf-tRNS)** for **cognitive enhancement** depends on the **task, target brain region, and individual variability**, but based on the literature, I would suggest:

Recommended Intensity Range:

- **1.5mA – 2.0mA** – Commonly used in cognitive studies with reported benefits.
- **1.0mA** – Used in some studies but less frequently tested for cognitive enhancement.

Supplementary Figure 6. The response to the prompt asking Gemini "I would like to use high frequency transcranial random noise stimulation for cognitive enhancement. What mA intensity do you suggest I will choose for stimulation?" yields a recommendation to use 1.5mA.

It's important to understand that transcranial random noise stimulation (tRNS) is still an area of active research, and optimal parameters, including intensity, can vary significantly depending on:

- **The specific cognitive function being targeted:** Different brain regions and cognitive processes may respond differently to varying intensities.
- **Individual variability:** Factors like age, brain anatomy, and individual sensitivity can influence the effects of tRNS.[1]

  1. Motor Task-Dependent Dissociated Effects of...
  www.frontiersin.org

- **Electrode placement and size:** These factors affect the distribution of the electrical current in the brain.

However, I can provide some general information based on current research:

- Research studies using high-frequency tRNS often utilize intensities in the range of 1 mA to 2 mA. 1.5mA appears to be a common intensity used in studies.
- It's crucial to prioritize safety. Exceeding certain intensity levels can increase the risk of adverse effects, such as skin irritation or, in rare cases, more serious complications.
- It is vital to consult with a medical professional before engaging in any form of brain stimulation.

Here are some key points to consider:

- **Safety First:** It is very important to consult with a qualified healthcare professional or researcher with expertise in tRNS before using this technique. Self-administration of tRNS can be risky.
- **Research Findings:** Many studies use intensities around 1.5 mA, but the optimal intensity can vary.
- **Individualized Approach:** The ideal intensity may need to be adjusted based on individual responses and the specific cognitive goals.

In summary, while 1.5mA is often used, I cannot give medical advice. Therefore, consulting a medical professional is necessary.

Ask Gemini

+ Deep Research Canvas

Supplementary Table 1. Mean (M) and standard deviation (SD) of side-effect ratings for the three stimulation conditions and inferential statistics. All side-effects were rated between 1 and 4 (1=none, 2=mild, 3=moderate, 4=strong). Using paired t-tests we compared the side effects in sham vs. pBO and sham vs. One-size. A t value of 0 with a p-value of 1 resulted from comparisons that are identical in two conditions after retaining only complete cases. Non-applicable (NA) values reflect the situation when side effect ratings are all rated with the same value in both conditions across participants, e.g. all data points are 1.

Size effect	Sham		pBO		One-size		Sham vs. pBO		Sham vs. One-size	
	M	SD	M	SD	M	*SD*	*t*	*P*	*t*	*p*
Itching	1.44	0.75	1.45	0.71	1.35	0.49	0	1	0.27	.79
Skin redness	1.12	0.41	1.06	0.24	1.06	0.25	-1.44	.16	0	1
Headache	1.12	0.54	1.03	0.17	1.13	0.43	-1.36	.18	1.14	.26
Neck pain	1.12	0.33	1.06	0.24	1.1	0.3	-0.81	.42	0	1
Scalp pain	1.09	0.38	1	0	1.06	0.36	-1.36	.18	0.44	.66
Burning Sensation	1	0	1.09	0.52	1.06	0.25	1	.33	1.44	.16
Metallic Taste	1.03	0.17	1	0	1.06	0.25	-1	.33	1	.33
Sleepiness	1.44	0.82	1.39	0.79	1.35	0.75	-0.2	.85	-0.62	.54
Trouble Concentrating	1.62	0.99	1.45	0.79	1.61	0.92	-0.73	.47	1.16	.25
Nervousness/ Anxiety	1	0	1.03	0.17	1.03	0.18	1	.33	1	.33
Unpleasant Sensation	1.12	0.54	1.18	0.58	1.13	0.43	0.49	.62	1.8	.083
Dizziness	1	0	1.12	0.55	1	0	1.28	.21	NA	NA
Nausea	1	0	1	0	1	0	NA	NA	NA	NA
Discomfort	1.26	0.67	1.27	0.76	1.13	0.43	0.3	.79	-0.57	.57
Visual Sensations	1	0	1	0	1.03	0.18	-1	.33	1	.33

Supplementary Movies 1

The movie shows a sample of trials from the Air Traffic Control Task. In this task, a static aircraft symbol appeared within each quadrant of a radar circle. In the “safe path” condition, the airplanes are all oriented in the same direction, which can be either clockwise or counterclockwise. In the “critical path” condition, which appears infrequently, one of the aircrafts is oriented in the opposite direction to the other 3. We encourage the reader to detect the infrequent event(s) in which one aircraft out of four deviates from the “safe path” condition, indicating an imminent collision.

Supplementary References

1 van Bueren, N. E. R., van der Ven, S. H. G., Hochman, S., Sella, F. & Cohen Kadosh, R. Human neuronal excitation/inhibition balance explains and predicts neurostimulation induced learning benefits. *PLOS Biology* **21**, e3002193 (2023). https://doi.org/10.1371/journal.pbio.3002193

2 Dakwar-Kawar, O. *et al.* Examining the Effect of Transcranial Electrical Stimulation and Cognitive Training on Processing Speed in Pediatric Attention Deficit Hyperactivity Disorder: A Pilot Study. *Front Hum Neurosci* **16**, 791478 (2022). https://doi.org/10.3389/fnhum.2022.791478

3 Battaglini, L., Contemori, G., Penzo, S. & Maniglia, M. tRNS effects on visual contrast detection. *Neuroscience Letters* **717**, 134696 (2020). https://doi.org/https://doi.org/10.1016/j.neulet.2019.134696

4 Pirulli, C., Fertonani, A. & Miniussi, C. On the Functional Equivalence of Electrodes in Transcranial Random Noise Stimulation. *Brain Stimulation: Basic, Translational, and Clinical Research in Neuromodulation* **9**, 621-622 (2016). https://doi.org/10.1016/j.brs.2016.04.005

5 Pirulli, C., Fertonani, A. & Miniussi, C. The Role of Timing in the Induction of Neuromodulation in Perceptual Learning by Transcranial Electric Stimulation. *Brain Stimulation: Basic, Translational, and Clinical Research in Neuromodulation* **6**, 683-689 (2013). https://doi.org/10.1016/j.brs.2012.12.005

6 Fertonani, A., Pirulli, C. & Miniussi, C. Random noise stimulation improves neuroplasticity in perceptual learning. *The Journal of neuroscience : the official journal of the Society for Neuroscience* **31**, 15416-15423 (2011). https://doi.org/10.1523/jneurosci.2002-11.2011

7 Contemori, G., Trotter, Y., Cottereau, B. R. & Maniglia, M. tRNS boosts perceptual learning in peripheral vision. *Neuropsychologia* **125**, 129-136 (2019). https://doi.org/https://doi.org/10.1016/j.neuropsychologia.2019.02.001

8 Penton, T. *et al.* Using High Frequency Transcranial Random Noise Stimulation to Modulate Face Memory Performance in Younger and Older Adults: Lessons Learnt From Mixed Findings. *Frontiers in Neuroscience* **12** (2018). https://doi.org/10.3389/fnins.2018.00863

9 Moret, B., Donato, R., Nucci, M., Cona, G. & Campana, G. Transcranial random noise stimulation (tRNS): a wide range of frequencies is needed for increasing cortical excitability. *Scientific Reports* **9**, 15150 (2019). https://doi.org/10.1038/s41598-019-51553-7

10 Lema, A., Carvalho, S., Fregni, F., Gonçalves, Ó. F. & Leite, J. The effects of direct current stimulation and random noise stimulation on attention networks. *Scientific Reports* **11**, 6201 (2021). https://doi.org/10.1038/s41598-021-85749-7

11 Contò, F. *et al.* Attention network modulation via tRNS correlates with attention gain. *eLife* **10**, e63782 (2021). https://doi.org/10.7554/eLife.63782

12 Wu, P.-J. *et al.* The distinct and potentially conflicting effects of tDCS and tRNS on brain connectivity, cortical inhibition, and visuospatial memory. *Frontiers in Human Neuroscience* **18** (2024). https://doi.org/10.3389/fnhum.2024.1415904

13 Silva, A. E., Mungalsingh, M., Raudzus, L. & Thompson, B. Peripheral reading improvement after non-invasive electrical stimulation of early visual areas. *Investigative Ophthalmology & Visual Science* **65**, 1004-1004 (2024).

14 Potok, W., Bächinger, M., van der Groen, O., Cretu, A. L. & Wenderoth, N. Transcranial Random Noise Stimulation Acutely Lowers the Response Threshold of Human Motor Circuits. *The Journal of neuroscience : the official journal of the Society for Neuroscience* **41**, 3842-3853 (2021). https://doi.org/10.1523/jneurosci.2961-20.2021
